# Novel Liposomal Adjuvants Enhance the Immune Response to Recombinant *Staphylococcus aureus* Antigens in Dairy Heifers

**DOI:** 10.1155/vmi/7845041

**Published:** 2026-09-23

**Authors:** Joaquin Cicotello, Camila Miotti, Guillermo Suarez Archilla, Ana Ines Molineri, Diego Aliprandi, Agostina Peña, Ivana Gabriela Reidel, Sonia Ricotti, María Virginia Zbrun, Lourdes Fraile Wirth, Marcelo Lisandro Signorini, Luis Fernando Calvinho, Carolina Melania Isabel Veaute, Cecilia María Camussone

**Affiliations:** ^1^ Dairy Chain Research Institute (INTA-CONICET), INTA Rafaela Experimental Station, Argentina. Route 34 Km 227, Rafaela (2300), Santa Fe, Argentina; ^2^ Laboratory of Experimental Immunology, School of Biochemistry and Biological Sciences, National University of Litoral, Ciudad Universitaria S3000ZAA, Santa Fe, Argentina; ^3^ Department of Immuno-Oncology, Beckman Research Institute of City of Hope, Duarte, California, USA, cityofhope.org

**Keywords:** dairy cattle, infectious diseases, new generation adjuvants, *Staphylococcus aureus*, veterinary vaccines

## Abstract

The development of effective adjuvant systems remains a critical challenge in veterinary vaccine design, particularly for infectious diseases causing significant economic losses in dairy cattle. This study evaluated the immunological profile induced by a mixture of recombinant antigens, formulated with cationic liposomes containing different combinations of *gemini* lipopeptide AG2‐C16, bovine‐specific CpG oligodeoxynucleotide, and *S. cerevisiae* mannans, in dairy heifers. Thirty‐five dairy heifers aged 8–10 months were randomly assigned to seven groups and immunized with recombinant *Staphylococcus aureus* antigens (α‐toxin, β‐toxin, clumping factor A, and fibronectin‐binding protein A) formulated with different adjuvant combinations. Animals received two primary doses 21 days apart, followed by a booster dose 8 months later. Humoral immune responses were assessed through ELISA measuring specific immunoglobulin G (IgG), IgG1, and IgG2 levels, while cytokine production was evaluated via whole‐blood assays. Results demonstrate that liposomal formulations combining AG2‐C16, CpG‐ODN, and mannans induce a significant immune response, in heifers, with IFN‐γ production. These findings provide valuable insights for the development of new vaccine strategies in cattle.

## 1. Introduction

The expansion of the dairy industry has led to a heightened risk of infectious diseases among cattle, which not only results in significant economic losses but can also pose public health concerns. For this reason, it is essential to reduce the impact of such infections and ensure the health of livestock [1]. Among the pathogens responsible for these infections, *Staphylococcus aureus* is one of the most prevalent pathogens associated with bovine intramammary infections (IMIs) worldwide [2]. *S. aureus* mastitis is characterized by its chronic, contagious nature and low cure rates following antimicrobial treatment, which favor its persistence within the herd [3], resulting in reduced milk yield and quality, increased somatic cell counts, and elevated culling rates, ultimately causing significant economic losses for the dairy industry. Given this burden, the development of novel vaccines, in conjunction with adjuvant systems that enhance vaccine efficacy against *S. aureus,* represents a valuable strategy to reduce reliance on antimicrobial treatment and improve udder health in dairy herds [1, 4].

In this sense, several antigens have been identified as candidates for subunit vaccination against *S. aureus* IMIs. Our group has focused on four proteins involved in different stages of an IMI: clumping factor A (ClfA), fibronectin‐binding protein A (FnbpA), α‐toxin (At), and β‐toxin (Bt). Briefly, ClfA mediates the interaction between bacteria and plasmatic fibrinogen, leading to bacterial cell clumping and favoring immune evasion. FnbpA is involved in the internalization of *S. aureus* in epithelial and phagocytic cells, while At and Bt are secreted proteins that induce damage in the host cells [5].

Vaccine adjuvants are essential for enhancing both the immune mechanism and the overall vaccine effectiveness. They can also reduce the amount of antigen required, contributing to lower vaccine production costs. In this context, liposomes have emerged as a promising platform for adjuvant formulation due to multiple advantages [6, 7].

In previous research work, we developed a series of liposomal formulations containing a combination of two immunostimulants: the novel *gemini* lipopeptide AG2‐C16 [8] and a murine‐specific CpG‐ODN. Immunizing mice with recombinant ClfA from *S. aureus* formulated with these components led to a robust humoral immune response, a predominantly Th1 cellular response, and the co‐induction of Th2 and Th17 profiles, as well as IFN‐γ^+^ cytotoxic T‐cells [9]. Subsequently, we obtained a new formulation based on liposomes supplemented with mannans (Man) derived from *Saccharomyces cerevisiae*. Mannosylation enhances the delivery of antigens to APCs and favors their activation by being recognized by specific receptors on the surface, which facilitates endocytosis and antigen presentation through the MHC I and MHC II pathways [10]. However, none of these formulations have been evaluated in a bovine model to date.

This study analyzed the immune response in dairy heifers induced by recombinant *S. aureus* antigens formulated with cationic liposomes in combination with AG2‐C16, CpG‐ODN, and Man.

## 2. Materials and Methods

### 2.1. Antigen Production

The coding sequences of *S. aureus* At, Bt, ClfA, and FnbpA were amplified from clones previously obtained in the laboratory in pET32a (Novagen, USA) [11, 12], using specific oligonucleotides (Genbiotech SRL) (Table 1). The sequences were cloned into expression vectors that do not encode fusion proteins (pET24a, pET28c, pET‐TEV, Novagen, USA; Figure S1) by *Nde*I/*Sal*I (ER0581/ER0641, Thermo Scientific) restriction for At, ClfA, FnbpA, and *Eco*RI/*Sal*I (ER0271/ER0641, Thermo Scientific) restriction for Bt. Escherichia *coli* BL21 (DE3) cells were transformed with the obtained constructs. For soluble expression of recombinant Bt (rBt), an additional transformation with the plasmid PGro7 (groES‐groEL chaperones) (Takara Bio Inc.) was required. Transformed cells were grown for 18 h at 37°C with shaking at 230 rpm in LB medium (L3022, Sigma‐Aldrich) supplemented with 0.2 mg/mL kanamycin (K105‐5, Genbiotech SRL) for rAt, rClfA, and rFnbpA, and LB medium supplemented with 0.2 mg/mL kanamycin and 0.2 mM chloramphenicol (K1020‐5, Genbiotech SRL) for rBt. Protein expression was induced under the following conditions: FnbpA‐pET24a and ClfA‐pET‐TEV: 15 mM lactose (0156‐17, Difco), incubation 37°C, 3 h; At‐pET24a: 15 mM lactose, incubation 25°C, ON; and Bt‐pET28c/PGro7, 2 mg/mL L‐arabinose (sc‐221794B, Santa Cruz Biotechnology Inc.) (for chaperone expression), 0.5 mM IPTG (V3955, Promega), incubation 20°C, ON. Recombinant antigens were purified with a nickel nitrilotriacetic acid column (R90101, Thermo Scientific), and the concentration was quantified by absorbance measurement at 280 nm using a spectrophotometer (NanoDrop 2000, Thermo Scientific). Purity was analyzed by 15% sodium dodecyl sulfate–polyacrylamide gel electrophoresis (SDS–PAGE) followed by Coomassie brilliant blue staining. Protein identity was further confirmed by Western blot, using anti‐6x‐His tag monoclonal antibody (Thermo Fisher Scientific) (Figure S2).

**TABLE 1:** Specific primers used for the amplification of antigen‐encoding genes.

Antigen	Sense	Sequence (5′ ⟶ 3′)	Restriction enzyme	T°m	Size (bp)
FnbpA	Forward	catatggGTGGCCAAAATAGCGGTAA	*NdeI*	58°C	410
	Reverse	gtcgacTGGTGGCACGATTGGAGG	*SalI*		

ClfA	Forward	catatgGAAAATAGTGTTACGCAATCT	*NdeI*	54°C	1570
	Reverse	gtcgacCTCTGGAATTGGTTCAATTTC	*SalI*		

β‐toxin	Forward	catatgGGAGTGATAATGATGGTG	*NdeI*	54°C	1012
	Reverse	gtcgacACTATAGGCTTTGATTGGG	*SalI*		

α‐toxin	Forward	gaattcGCAGATTCTGATATTAAT	*EcoRI*	55°C	897
	Reverse	gtcgacATTTGTCATTTCTTCTTT	*SalI*		

*Note:* References: Lowercase sequences correspond to restriction enzyme recognition sites used for cloning. The table also includes annealing temperatures and expected PCR product sizes.

### 2.2. Liposomes and Immunostimulants

Cationic liposomes (Lip) were obtained using dipalmitoylphosphatidylcholine (Avanti Polar Lipids, Alabaster, AL, USA) and cholesterol and stearylamine (Sigma‐Aldrich, St. Louis, MO, USA) by the ethanolic injection method [13]. Briefly, the three components in 7:2:2 mol/mol ratio were dissolved in an ethanol/isopropanol mixture (1:1 v/v) (organic phase). The aqueous phase consisted of 50 mM sodium acetate buffer (BAc), pH 4.3. The organic phase was incorporated by injection to the aqueous phase in a 1:9 v/v ratio, resulting in a liposomal suspension with a lipid concentration of 16 mM. As immunostimulants (ISs), the bovine‐specific CpG oligonucleotide (CpG‐ODN) 5′‐tcgtcgtttgtcgttttgtcgtt‐3′ [14] with phosphodiester linkages (synthesized by Genbiotech SRL), the *Gemini* lipopeptide (AG2‐C16) [8, 9], synthesized by the research group of the Departamento de Química Orgánica of the FBCB‐UNL, and/or commercial Man from *S. cerevisiae* (Sigma‐Aldrich, St. Louis, MO, USA) were used.

### 2.3. Formulation of Immunogens

The experimental multicomponent immunogens consisted of a mixture of each of the recombinant *S. aureus* antigens rFnbpA, rClfA, rBt, and rAt obtained (Section 2.1) (Rec), formulated with Lip (Section 2.2) and different combinations of ISs as follows: Lip + CpG‐ODN + Rec, Lip + AG2‐C16 + Rec, Lip + CpG‐ODN + AG2‐C16 + Rec, Lip + Man + Rec, Lip + CpG‐ODN + AG2‐C16, and Lip + Man. The final concentrations of each component in the formulations were Lip 4 mM; CpG‐ODN 7.5 nmol/mL; AG2‐C16 400 μM; Man 2 mg/mL; and Rec 100 μg each/dose. The formulations were incubated at 4°C for 72 h with periodic observation of their stability (absence of precipitate or lumps). Twenty‐four hours after their preparation, they were analyzed by dynamic light scattering (DLS) using a Zetasizer (Malvern Instruments Ltd., Malvern, UK) to determine the predominant size (hydrodynamic diameter, D) as well as the size dispersion of the liposomes in suspension (polydispersity index, PDI). The suspension was equilibrated for 3 min at 25°C prior to measurement. PDI values below 0.5 are associated with uniform particle sizes [15]. Size distribution results were obtained by averaging 3 consecutive measurements, each consisting of 14 runs of 15 s.

### 2.4. Animals and Immunizations

Thirty‐five heifers, aged between 8 and 10 months, belonging to the dairy herd of the INTA Rafaela Experimental Station (Santa Fe, Argentina) were selected and randomly divided into 7 groups. The vaccinated groups were G1) Lip + CpG‐ODN + Rec (*n* = 5); G2) Lip + AG2‐C16 + Rec (*n* = 5); G3) Lip + CpG‐ODN + AG2‐C16 + Rec (*n* = 5); and G4) Lip + Man + Rec (*n* = 5), while the control groups were C1) unimmunized control (*n* = 5); C2) Lip + CpG‐ODN + AG2‐C16 (*n* = 5, as vehicle control for G1, G2, and G3); and C3) Lip + Man (*n* = 5, as vehicle control for G4). The vehicle controls Lip + CpG‐ODN and Lip + AG2‐C16 were previously assessed and were found to induce no immune response [9, 13]. In order to reduce the number of animals used in this study, only the vehicle control containing both immunostimulants was included. Two doses of 2 mL immunogen or controls were administered subcutaneously in the neck [13] at 0 and 21 days. Eight months after the initiation of the immunization plan, a booster dose was applied subcutaneously in the neck on a total of 34 heifers (G1: *n* = 5; G2: *n* = 5; G3: *n* = 5; G4: *n* = 5; C1: *n* = 5; C2: *n* = 4; and C3: *n* = 5). One nonpregnant animal belonging to C2 was transferred to another herd and became unavailable for further study.

### 2.5. Blood Sampling

Periodic blood sampling was performed by coccygeal vein puncture of all animals approximately every 15 days from Day 0 (first dose) to 120, and for 45 days after the booster administration. Blood samples were allowed to clot at room temperature and then centrifuged. The resulting sera were stored at −20°C until required. Twenty‐one days after the second vaccine or control dose, heparinized blood samples were obtained by jugular vein puncture for the determination of cytokine production. Samples were used within 4 h after collection. Animals were monitored for adverse reactions following each immunization (swelling, local heat, tenderness). A graphic description of the study is available as Supporting information (Figure S3).

### 2.6. Evaluation of Immune Response

#### 2.6.1. Humoral Immune Response

Indirect ELISA tests were performed to determine serum antibody responses (IgG, IgG1, and IgG2) against rFnbpA, rClfA, rAt, and rBt according to Reidel et al. [13]. Briefly, 96‐well flat‐bottom plates (655061, Greiner Bio‐One, Frickenhausen, Germany) were coated with 0.5 μg of each protein per well, dissolved in sodium carbonate buffer (pH 9.6), and incubated for 2 h at 37°C. Plates were first incubated with a 5% (w/v) solution of low‐fat goat milk powder dissolved in PBS at pH 7.4 and then with heifer sera diluted in a 1% (w/v) solution of low‐fat goat milk powder dissolved in PBS at pH 7.4. Next, the wells were incubated with antibovine IgG (A5295, Sigma‐Aldrich) or antibovine IgG1 antibodies (PA1‐84658, BioRad Laboratories, CA, USA), both conjugated with horseradish peroxidase, or mouse antibovine IgG2 (B8400, Sigma‐Aldrich) followed by horseradish peroxidase conjugated goat antimouse IgG (A4416, Sigma‐Aldrich). All incubations were performed for 1 h at 37°C. After each step, plates were washed three times with a 0.05% solution of Tween 20 (2003‐07, Biopack) in PBS. Finally, H_2_O_2_/tetramethylbenzidine enzyme substrate (TMB; 002023, Thermo Scientific) was added, and the color reaction was stopped by the addition of 0.5 N H_2_SO_4_. Optical density (OD) was measured at 450 nm using a plate reader (Infinite F50; Tecan Trading AG, Switzerland). Antibody levels were expressed in terms of OD or titer.

#### 2.6.2. Cytokine Production

Antigen‐specific whole‐blood assays (WBAs) were performed as described by Rainard et al. [16]. Briefly, 1.5 mL blood was added to 24‐well cell culture plates (07‐6024, Biologix Group Ltd) in the presence of 500 μL of culture medium (RPMI‐1640; 21870076, Thermo Scientific) as negative control, 500 μL of RPMI‐1640 supplemented with 10 μg/well of pokeweed mitogen (PWM; L8777, Sigma‐Aldrich) as nonspecific stimulus, and RPMI‐1640 supplemented with 25 μg/well of rClfA or rFnbpA as specific stimuli. Mixtures were cultured in 5% CO_2_ atmosphere at 37°C for 48 h, and then, supernatants were collected and stored at −80°C until use. The concentration of IFN‐γ, IL‐4, IL‐5, and IL‐17 was determined by ELISA with commercial kits (3119‐1H‐6, 3118‐1H‐6, 3191‐1H‐6, and 3109‐1H‐6, respectively, MAbtech AB, NackaStrand, Sweden).

### 2.7. Statistical Analysis

For repeated measurements over time, the Friedman test was applied to compare the four immunized groups with the nonimmunized control, as well as to compare G1, G2, and G3 with C2. The Wilcoxon test was used to compare G4 with C3. Statistical differences between G1, G2, and G3 with C2 at specific time points were analyzed using the Kruskal–Wallis test, followed by Dunn’s correction for multiple comparisons. The Mann–Whitney test was used to compare the G4 with C3. Statistical significance was set at *p* < 0.05, and all tests were two‐tailed. Results are presented as median ± interquartile range (IQR) for each group. All statistical analyses were performed using GraphPad Prism Version 8.0.1 for Windows (GraphPad Software, San Diego, CA, USA).

### 2.8. Ethical Statement

All procedures were consistent with the Guide for the Care and Use of Agricultural Animals in Agricultural Research and Teaching [17] and were approved by the Comité de Ética y Seguridad del Centro Regional Santa Fe del Instituto Nacional de Tecnología Agropecuaria (CICUAE, N°21‐0120; June 6, 2021) and by the Comité de Ética y Seguridad en la Investigación de la Facultad de Bioquímica y Ciencias Biológicas de la Universidad Nacional del Litoral (CE2021‐35; March 16, 2021).

## 3. Results

### 3.1. Formulations

The particle size was measured by DLS for the different formulations (Table 2). The D of empty liposomes were 201.2 nm, which increased after the addition of other components.

**TABLE 2:** Hydrodynamic diameter and polydispersity index (PDI) of liposomal formulations as determined by dynamic light scattering.

Liposomal formulations	D (nm) ± SD	PDI
Liposomes	201.2 ± 52,64	0.224
Lip + CpG‐ODN	741.1 ± 246.2	0.325
Lip + CpG‐ODN + Rec	1741 ± 283.9	0.638
Lip + AG2‐C16	254.8 ± 94.47	0.385
Lip + AG2‐C16 + Rec	221.8 ± 59.15	0.263
Lip + AG2‐C16 + CpG‐ODN	266.6 ± 84.62	0.297
Lip + AG2‐C16 + CpG‐ODN + Rec	225.6 ± 58.92	0.379
Lip + Man	207.8 ± 51.03	0.311
Lip + Man + Rec	211.5 ± 50.47	0.233

*Note:* References: D: hydrodynamic diameter (mean of three measurements).

The addition of CpG‐ODN increased both the D of the particles (from 201.2 nm to 741.1 nm, 3.7‐fold increase) and the particle dispersity (PDI values from 0.224 to 0.325, 45% increase). In contrast, the incorporation of AG2‐C16 or Man (in Lip + AG2‐C16, and Lip + AG2‐C16 + Rec, or Lip + Man and Lip + Man + Rec, respectively) resulted in a slight increase in the particle D (254.8 nm, 221.8 nm, 207.8 nm, and 211.5 nm, respectively), compared with empty liposomes, with little changes in particle homogeneity (PDI values of 0.385, 0.263, 0.311, and 0.233, respectively).

In turn, the addition of AG2‐C16 to the mixtures containing CpG‐ODN (Lip + CpG‐ODN + AG2‐C16 and Lip + CpG‐ODN + AG2‐C16 + Rec) appears to stabilize the formulations, generating lower size dispersity (PDI values of 0.297 and 0.379, respectively) and smaller diameter (266.6 nm and 225.6 nm, respectively) compared with the formulations containing only CpG‐ODN (741.1 nm, PDI = 0.325 for Lip + CpG‐ODN and 1741 nm, PDI = 0.638 for Lip + CpG‐ODN + Rec). The incorporation of Rec had minimal effect on the particle size of the AG2‐C16 and Man supplemented liposomes (221.8 nm and 211.5 nm, respectively).

### 3.2. Humoral Immune Response

#### 3.2.1. IgG Anti‐Rec in Serum

**FIGURE 1:**
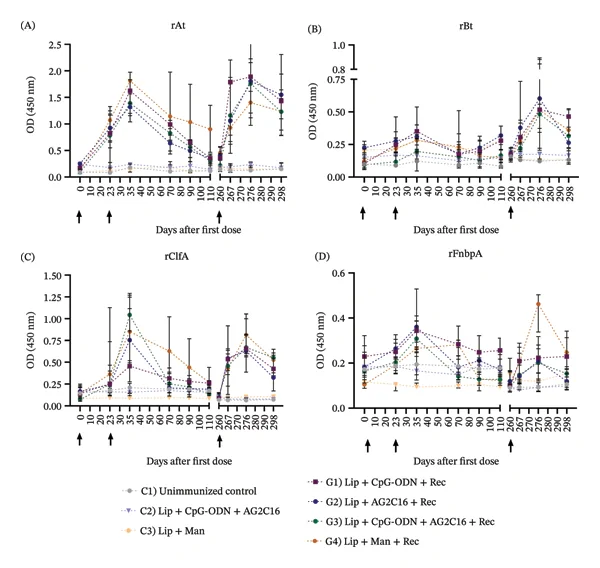
Specific IgG levels in serum from heifers immunized with different formulations containing four recombinant antigens ((A) anti‐rAt, (B) anti‐rBt, (C) anti‐rClfA, and (D) anti‐rFnbpA), measured by indirect ELISA. The immunization schedule consisted of two doses and a booster. Sera were diluted 1:1000. Optical density (OD) was measured at 450 nm. Data are presented as median ± interquartile range (IQR) for each group. Arrows indicate the days of vaccination.

The sera of immunized animals specifically recognized all four recombinant antigens (Figure S2). Temporal comparisons were made using the Friedman or Wilcoxon test as appropriate (see Methods 2.7). Increases in IgG levels over time were observed in all immunized groups (Figure 1; Figure S4). Compared to the unimmunized control (C1), Group G1 showed significantly higher IgG levels for all four recombinant antigens, while G2 exhibited significant increases for rAt (*p* < 0.05) and rBt (*p* < 0.05), G3 for rClfA (*p* < 0.05), and G4 for rAt (*p* < 0.05), rBt (*p* < 0.05), and rClfA (*p* < 0.05). When compared to vehicle controls, the immunized groups also exhibited significantly higher levels, with some exceptions. Relative to C2, the G1 group exhibited significant increases for all antigens (*p* < 0.05), the G2 group showed significant differences for rAt (*p* < 0.01), rBt (*p* < 0.01), and rClfA (*p* < 0.05), while the G3 group displayed significant increases only for rClfA (*p* < 0.05). Compared to C3, G4 showed significant differences for rAt (*p* < 0.01), rClfA (*p* < 0.001), and rFnbpA (*p* < 0.05). No significant differences in IgG levels for the four vaccine antigens were observed among the different immunogenic formulations throughout the trial (*p* > 0.12).

Individual time point analysis was performed using the Kruskal–Wallis or Mann–Whitney test as appropriate (see Methods 2.7). Results demonstrated that after two doses, vaccination generated an increase in antigen‐specific IgG levels in all the immunized groups compared to controls. These levels reached their maximum at approximately Day 35 from the start of the trial (Figure 1; Figure S4). At Day 35, Group G1 showed significant increases for all four antigens (rAt and rBt, *p* < 0.01; rClfA and rFnbpA, *p* < 0.05), whereas G2 displayed significant differences for rAt, rClfA, and rFnbpA (*p* < 0.05). G3 showed significant responses for rAt (*p* < 0.05) and rClfA (*p* < 0.01), while G4 showed significant increases for rAt, rBt, and rClfA (*p* < 0.01).

A booster was administered approximately 8 months (Day 260) after initiating the immunization schedule (Figure 1). Following booster vaccination, anti‐Rec IgG reached their maximum levels at 15 days after booster administration (Day 276). At Day 276, Groups G1 and G4 exhibited the broadest responses with significant increases for all four recombinant antigens (*p* < 0.05), whereas in groups G2 and G3, significant increases were mainly detected for rAt, rBt, and rClfA (*p* < 0.05).

The complete statistical comparisons for each time point are provided in Table S1.

No specific IgG was detected in preimmune serum from any of the animals included in the study, and no adverse reaction at the injection site was observed in any group.

#### 3.2.2. IgG Titers

**FIGURE 2:**
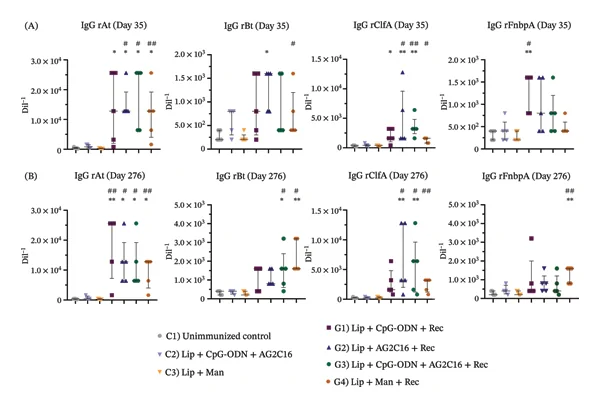
IgG antibody titers against rAt, rBt, rClfA, and rFnbpA in serum from heifers after two doses (Day 35) (A) and 15 days after the booster immunization (Day 276) (B). The sera were serially diluted from 1:200 to 1:25600. The cut‐off value was then calculated as the mean plus three standard deviations of the optical density (OD) obtained for the respective control. Data are presented as median ± interquartile range (IQR). Statistical analysis was performed using the Kruskal–Wallis test followed by Dunn’s multiple comparisons test. ^∗^ *p* < 0.05, ^∗∗^ *p* < 0.01, ^∗∗∗^ *p* < 0.001, ^∗∗∗∗^ *p* < 0.0001 vs. unimmunized control; #*p* < 0.05, ##*p* < 0.01, ###*p* < 0.001, ####*p* < 0.0001 vs. vehicle control (C2 for G1, G2, and G3 and C3 from G4)).

IgG titers were evaluated at the points of maximal response identified in the kinetic analysis (3.2.1), both after the initial immunization schedule (Day 35) and following the booster administration (Day 276) (Figure 2).

After the second dose on Day 35, all immunized groups showed increased specific antibody titers compared to controls. Groups G2 and G3 elicited significant IgG responses against rAt and rClfA versus both controls (C1 and C2), with G2 additionally responding to rBt (versus C1 only). Group G1 induced responses against rAt and rClfA (versus C1 only) and rFnbpA (versus both controls). Group G4 induced significant responses against rAt (versus both C1 and C3), rBt, and rClfA (versus C3 only).

Fifteen days after the booster immunization (Day 276), antibody levels increased to values similar to those observed on Day 35 following the initial two‐dose schedule. Group G4 showed the broadest response, inducing significant antibody titers against all antigens (rAt, rBt, and rFnbpA versus C1 and C3 and rClfA versus C3 only). Groups G1, G2, and G3 elicited significant IgG responses against rAt versus both controls (C1 and C2), with G2 additionally responding to rClfA (versus both controls) and G3 to rBt and rClfA (versus both controls).

#### 3.2.3. Anti‐Rec IgG1 and IgG2 in Serum

**FIGURE 3:**
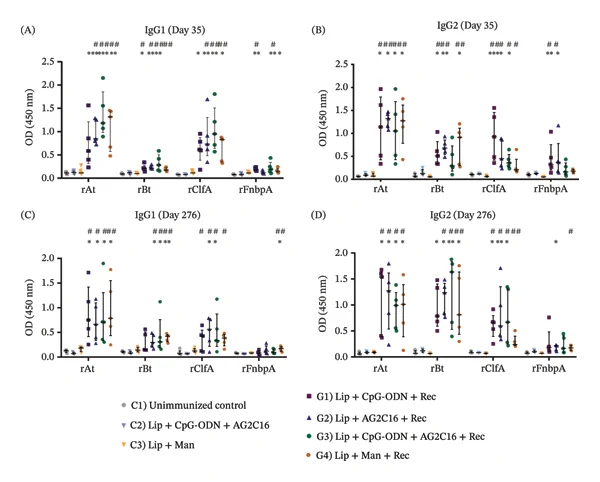
Levels of IgG1 (1:1000 dilution) and IgG2 (1:500 dilution) antibodies against rAt, rBt, rClfA, and rFnbpA in heifer’s serum. (A) IgG1 after two doses (Day 35), (B) IgG2 after two doses (Day 35), (C) IgG1 15 days after the booster immunization (Day 276), and (D) IgG2 15 days after the booster immunization (Day 276). Data are presented as median ± interquartile range (IQR). Statistical analysis was conducted using the Kruskal–Wallis test followed by Dunn’s multiple comparisons test (^∗^ *p* < 0.05, ^∗∗^ *p* < 0.01, ^∗∗∗^ *p* < 0.001, ^∗∗∗∗^ *p* < 0.0001 vs. unimmunized control. #*p* < 0.05, ##*p* < 0.01, ###*p* < 0.001 vs. vehicle control (C2 from G1, G2, and G3 and C3 from G4)).

After two doses, all experimental formulations induced the production of specific IgG1 antibodies (Figure 3A). Group G4 showed significant increases against all antigens (rAt and rClfA versus both controls, rBt versus C3 only, and rFnbpA versus C1 only). Groups G2 and G3 elicited significantly elevated IgG1 levels against rAt, rBt, and rClfA versus both controls (C1 and C2), with G2 additionally responding to rFnbpA (versus both controls). Group G1 induced significant responses against rBt and rFnbpA (versus both controls), and rAt and rClfA (versus C1 only).

Fifteen days after the booster dose, all four formulations maintained IgG1 production at levels comparable to Day 35 (Figure 3C). Group G4 enhanced its response breadth, now showing significance against rAt, rBt, and rFnbpA versus both controls (C1 and C3), and rClfA versus C3 only. IgG1 levels were significantly higher in Groups G2 and G3 against rAt, rBt, and rClfA compared to both controls (C1 and C2). Group G1 exhibited significant differences only for rAt (versus both controls) and rClfA (versus C2 only).

Regarding IgG2 levels (Figure 3B), after two doses of immunogens, all formulations induced the production of this subclass. Groups G1 and G2 elicited significantly elevated IgG2 levels against all four antigens (rAt, rBt, rClfA, and rFnbpA) versus both controls (C1 and C2). Group G3 showed significant increases for rAt and rClfA (versus both controls), while Group G4 exhibited significant differences against rAt and rBt (versus both C1 and C3), and rClfA (versus C3 only).

Fifteen days after the booster dose (Day 276), the IgG2 response patterns shifted notably. Groups G2 and G3 maintained their responses against rAt, rBt, and rClfA versus both controls. Group G1 showed a reduced profile compared to Day 35, with significant increases only against rAt and rClfA (versus both controls) and rBt (versus C1 only). In contrast, Group G4 enhanced its response breadth, now showing significant elevation against all four antigens (rAt and rBt versus both C1 and C3, and rClfA and rFnbpA versus C3 only).

### 3.3. Cytokine Production

Levels of interferon‐γ (IFN‐γ), IL‐17, IL‐5, and IL‐4 were quantified in whole‐blood culture supernatant, 21 days after the second dose, following stimulation with rFnbpA and rClfA.

**FIGURE 4:**
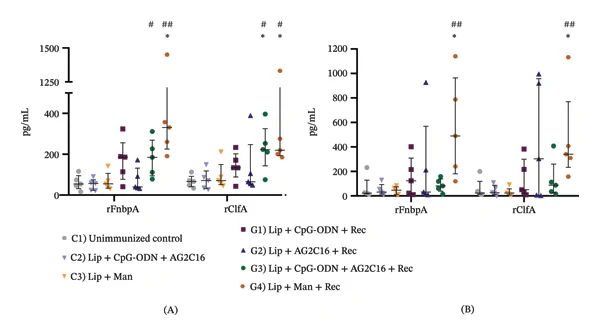
Whole blood assay stimulated with rFnbpA and rClfA. (A) IFN‐γ production. (B) IL‐17A production. Blood from heifers was collected 21 days after the second immunization and cultured for 48 h in 24‐well plates in the presence of 25 μg/well of rFnbpA and rClfA. Results are shown as median ± IQR. Statistical analysis was performed using the Kruskal–Wallis test followed by Dunn’s multiple comparison test (^∗^ *p* < 0.05, ^∗∗^ *p* < 0.01, ^∗∗∗^ *p* < 0.001, compared with unimmunized control. #*p* < 0.05, ##*p* < 0.01, ###*p* < 0.001 vs. vehicle control (C2 from G1, G2, and G3 and C3 from G4)).

Both stimuli induced IFN‐γ production in all immunized groups (Figure 4A). Group G4 showed significantly elevated IFN‐γ levels in response to both antigens (*p* < 0.05), compared to both controls (C1 and C3). G3 exhibited significantly higher IFN‐γ production for rClfA compared to C1 and C2, and rFnbpA compared only to C2.

Regarding IL‐17, significant increases were observed only in Group G4 in response to both rFnbpA and rClfA, compared to both controls (C1 and C3).

No differences in IFN‐γ or IL‐17 production were detected among the immunized groups. In addition, no production of IL‐4 or IL‐5 was detected in any of the groups following stimulation with either antigen.

Table 3 summarizes the characteristics of the two most remarkable formulations.

**TABLE 3:** Summary of the main characteristics of the two most remarkable formulations.

Formulation	Particle size (nm)/PDI	Key immune features	Predominant profile
G3) Lip + CpG‐ODN + AG2‐C16 + Rec	225.6/0.379	• IgG response to 3/4 antigens postbooster (rAt, rBt, rClfA) • IgG1/IgG2: 3/4 antigens (IgG2 broadens postbooster). • Significant IFNγ increase (rClfA vs both controls; rFnbpA vs C2 only)	Th1

G4) Lip + Man + Rec	211.5/0.233	• IgG response to 4/4 antigens postbooster (rAt, rBt, rClfA, rFnbpA) • IgG1/IgG2: 4/4 antigens (IgG2 broadens postbooster) • Significant IFNγ and IL‐17 (both antigens vs both controls).	Th1/Th17

## 4. Discussion

Subunit vaccines represent a promising approach in veterinary immunization, allowing standardization of the product, minimizing the manipulation of pathogens and increasing the safety profile of the immunogen. Together with the antigen design, the development of effective adjuvant systems represents a fundamental challenge in modern veterinary vaccinology. Recently, our group developed novel liposomal formulations containing combinations of AG2‐C16, bovine‐specific CpG‐ODN, and *S. cerevisiae* Man as immunostimulants. To evaluate their adjuvant potential, we used recombinant *S. aureus* antigens as an antigenic model, given that this pathogen constitutes an important problem in animal health. In the present study, we characterized the immune response induced by these adjuvant formulations in dairy heifers. This work aims to assess, for the first time, the effect of using several adjuvant formulations to formulate the described antigens on the immune response of calves. As an initial approach, we studied the systemic immune response following subcutaneous administration. The immunization scheme in this study targets young heifers since *S. aureus* can cause IMIs even before first calving [18–20]. Vaccination of this age segment has been proposed in the literature as a complementary strategy to reduce this risk, as heifers are excluded from traditional mastitis control measures. As immunization starts before lactation onset, milk sampling was not applicable; serum was therefore evaluated as the systemic compartment reflecting the subcutaneous route used.

The liposome‐based adjuvants presented here are designed to act both as a delivery system and as an immune activator. As a delivery system, liposomes protect antigen integrity and enhance co‐delivery of the antigen and immunostimulants to antigen‐presenting cells (APCs), a process influenced by particle size, which determines cellular uptake efficiency. As an immune activator, the immunostimulants embedded within the liposomal formulation engage distinct innate immune pathways upon uptake, shaping the resulting adaptive immune profile. In this regard, particle size assessment provides data on the ability of these formulations to perform these functions. DLS measurements indicated that the hydrodynamic diameter of the majority of particles in the formulations was approximately 200 nm. It has been demonstrated that particles with a size range from 20 to 200 nm induce more potent responses than those of larger particles [21]. Particles measuring less than 500 nm have been shown to exhibit enhanced biodistribution and uptake by APCs. The Th profile can also be influenced by the size and shape of the nanoparticles. For example, spherical particles measuring approximately 200 nm have been associated with Th1 and Th2 profiles [22]. Furthermore, the measurement of size in conjunction with PDI, as an indicator of particle size uniformity [15], provides an assessment of variation between batches. This is a necessary component for standardizing the preparation method.

The inclusion of the various immunostimulants evaluated in this study affected the hydrodynamic diameter of liposome particles in different ways. As we had previously observed, the addition of CpG‐ODN to liposomes generated an increase in size compared with empty liposomes along with an increase in the PDI value, which was associated with a tendency toward aggregation [9]. This effect was more marked in the presence of Rec antigens. Increased PDI and hydrodynamic diameter suggest interactions between the cationic lipid bilayer and negative CpG‐ODN charges. This potentially leads to the formation of multilamellar vesicles and particle aggregates [23].

However, the incorporation of AG2‐C16 into this formulation appeared to have a stabilizing effect by reducing the PDI of the Lip + CpG‐ODN + AG2‐C16 formulation compared with Lip + CpG‐ODN, with and without protein addition. This effect could be associated with charge compensation due to the cationic nature of AG2‐C16 [24]. Regarding the incorporation of Man into empty liposomes, a slight increase in hydrodynamic diameter was recorded. This finding suggests an effective interaction between Man and liposomes, without evidence of a tendency toward the formation of visible precipitates, as confirmed by the low PDI values. Similar results were reported for liposomes containing mannosylated cholesterol [25], where no significant differences in size and zeta potential were observed between liposomes with and without mannose. Additionally, Goswami et al. [26] evaluated the incorporation of mannan‐cholesterol conjugates with different mannan lengths (mono‐ to tetrasaccharides) and observed that, although this incorporation increased both diameter and PDI, it remained at values below 0.5, and this effect was diminished by adding 0.3%–2% PEG to the formulations.

In this study, these adjuvants were employed to formulate a mixture of recombinant molecules corresponding to *S. aureus* virulence factors (rAt, rBt, rClfA, and rFnbpA). In previous studies, antibodies raised in heifers against these recombinant antigens significantly reduced bacterial adherence to fibronectin and fibrinogen, inhibited *S. aureus* invasion of mammary epithelial cells, reduced hemolytic activity, and enhanced neutrophil phagocytosis, in vitro [12, 27]. Given this evidence, we considered these antigens as an appropriate model system to evaluate our novel adjuvant formulations. In the former studies, antigens were obtained as thioredoxin (TRX) fusion proteins (pET32a, Novagen, USA) [11–13, 27]. Given that TRX has the potential to be immunogenic by itself [28], in the present study, we implemented a cloning strategy that produces recombinant proteins without fusion tags. This allows us to target the immune response more precisely to each antigen. However, we observed a notably reduced humoral response against rFnbpA compared to the other vaccine antigens (rAt, rBt, and rClfA) and also compared to the same antigen fused to TRX evaluated previously [13]. The absence of TRX may have resulted in an unstable conformation or aggregate formation with reduced epitope exposure, thereby compromising B‐cell activation. This issue affected rFnbpA more severely than the other antigens, suggesting that rFnbpA may be structurally more susceptible to folding and stability problems without TRX. Conversely, rFnbpA was able to stimulate a cellular response, which reinforces the idea of deficient conformational epitope availability.

The humoral immune response, induced by the formulations in dairy heifers, was analyzed. After two doses, all formulations were found to induce the production of specific IgG antibodies against the four vaccine antigens. These results suggest that the two‐dose vaccination schedule was effective in inducing a humoral response against most vaccine antigens, with antibody levels remaining elevated for more than a month, especially for rAt, rBt, and rClfA. These findings are consistent with those previously reported by our group [13], where it was observed that a two‐dose schedule with a formulation containing Lip + CpG‐ODN as an adjuvant was sufficient to stimulate the production of specific antibodies in heifers. Likewise, it was evidenced that the inclusion of a third dose in the initial schedule had a limited effect on the stimulation of the humoral immune response [13]. Novoa et al. [29] demonstrated that the immunization of steers with two doses of a recombinant vaccine formulated with four *Neospora caninum* proteins and Lip + CpG‐ODN stimulated significant levels of *anti-N. caninum* IgG antibodies, with values above the cut‐off point from Day 21 that were maintained until Day 84 after the first dose. Saravanan et al. [30] evaluated the efficacy of a foot‐and‐mouth disease vaccine formulated with an oil‐emulsion‐based liposomal adjuvant (VacciMax) combined with CpG‐ODN in calves aged 6–12 months. Animals were immunized with an initial dose, followed by a booster at 6 months. Virus neutralization test (VNT) antibody titers reached significant levels between 14 and 28 days postvaccination, depending on the serotype evaluated. The vaccine provided 25% protection 1 year after a single dose, a percentage that increased to 86% after a booster administration, highlighting the importance of implementing a booster vaccination schedule to optimize protective efficacy. In the present study, although protection evaluation was beyond the scope of this research work, a booster administration 8 months after initiating the immunization plan stimulated antibody production to levels that were similar to or higher than those achieved after the second dose in all immunized groups, suggesting the formulations’ ability to generate immunological memory. The similarity of antibody levels postbooster to those on Day 35 may reflect the fact that the initial two‐dose schedule elicits nearly the maximum response to the antigens and adjuvants used. Furthermore, these results support the implementation of a simple immunization schedule that can be easily adjusted to fit the animals’ regular management routines. Similar results were observed in previous works by our group [13].

To our knowledge, there are no previous reports on the use of liposomes formulated with *gemini*‐type lipopeptides or Man in cattle. However, some studies have explored similar formulations. Nishimura et al. [31] demonstrated that the immunization of male calves with two doses of a recombinant *N. caninum* antigen, formulated with oligomannose‐coated liposomes, induced the production of specific serum antibodies 15 days after the second dose. On the other hand, Kornuta et al. [32] evaluated a gene vaccine against bovine herpesvirus Type 1 formulated with mannosylated liposomes in cattle, observing the production of specific antibodies after two vaccine doses and a booster 60 days after the first vaccination. However, differences in immunization schedules and the type of vaccine antigen used make direct comparison with our results difficult. The findings in this study underscore the adjuvant capacity of both Man and *gemini*‐type lipopeptides, particularly AG2‐C16, in combination with liposomes, demonstrating their ability to potentiate the immune response in this animal model.

One of the main functions of adjuvants is to stimulate a specific immunological profile. Regarding the evaluation of IgG subclasses in blood, in this study, we observed that all vaccine formulations stimulated the production of specific IgG1 and IgG2 against the four antigens evaluated. In particular, after the second dose (Day 35) and postbooster (Day 276), the Lip + CpG‐ODN + Rec, Lip + CpG‐ODN + AG2‐C16 + Rec, and Lip + AG2‐C16 + Rec formulations promoted a significant increase in IgG1 for most antigens, while the Lip + Man + Rec formulation induced a robust IgG1 response against all of them. All formulations significantly increased IgG2 levels, followed by an additional increase postbooster in all groups. However, the magnitude of the response varied depending on the antigen and formulation. These results suggest that adjuvant formulations modulate IgG2‐associated responses in cattle. The administration of recombinant *N. caninum* proteins formulated with Lip + CpG‐ODN to steers [29] induced elevated levels of specific IgG1 against the four antigens used and significantly higher levels of specific IgG2 against three antigens, 7 days after the second dose compared to control groups. The differential responses observed between antigens in the present study may stem from variations in both antigenic properties and adjuvant formulations, although determining the specific contribution of each component was beyond the scope of this study.

Protective immunity depends not only on antibody production but also on the activation of the cellular immune response. In particular, Th1 and Th17 profiles have been shown to play an important role in controlling *S. aureus* infection [33, 34]. Therefore, characterizing the cytokine profile stimulated by the adjuvants may provide valuable insights into functional aspects of the formulations. Our results suggest that immunization with the tested formulations predominantly favored a Th1 response, evidenced by the increase in IFN‐γ production in all immunized groups compared with controls, with a significant effect in the Lip + CpG‐ODN + AG2‐C16 + Rec and Lip + Man + Rec groups, suggesting a greater potential of these formulations to induce a Th1‐associated cytokine profile. The production of IgG2 in response to these formulations is consistent with the induction of the Th1 profile. It has been described that CpG‐ODN exerts an immunostimulatory action inducing T‐cell differentiation to the Th1 profile [35]. However, the structure of the CpG‐ODN given by its sequence and the type of linkages can induce different responses depending on the species [36]. The CpG‐ODN used in this study has phosphodiester linkages throughout their entire backbone. To the best of our knowledge, this is the first report of the effect of this type of CpG‐ODN on the cellular response in cattle. Additionally, the administration of Lip + Man + Rec was associated with the production of IL‐17. On the other hand, the absence of detectable production of IL‐4 and IL‐5 suggests that the evaluated formulations did not promote a significant Th2 response. This finding is consistent with the expected immunological behavior of CpG‐ODN and cationic liposomal adjuvants, which favor Th1‐type responses and IFN‐γ production. However, methodological factors must also be considered. IL‐4 is typically produced at relatively low concentrations and exhibits transient kinetics depending on the antigen, species, and stimulation conditions. Since cytokines were quantified in WBA supernatants at 48 h poststimulation, it is possible that IL‐4 and IL‐5 production occurred earlier or remained below the detection threshold at the selected time point. Since IL‐4 is often produced at low levels and has been described as exhibiting transient kinetics [37], future studies with additional measurements may further clarify Th2‐associated responses. Taken together, these findings reinforce the ability of the formulations to induce a predominantly Th1 cytokine profile with a contribution of Th17 in the presence of Man. It is important to note that the induction of specific IgG, IgG subclasses, or Th1‐associated cytokines does not necessarily equate to clinically meaningful protection against natural *S. aureus* infection. Immune correlates of protection against bovine *S. aureus* mastitis remain poorly defined, and no validated threshold of antibody or cytokine response has been established as predictive of clinical outcome. Some evidence suggests that specific antibody isotypes may contribute to functional protection, as IgG2 has been shown to be opsonic for bovine neutrophils, whereas IgG1 can inhibit this activity [38], but these associations remain incompletely understood. Therefore, while the humoral and cytokine responses observed in this study are compatible with a Th1‐biased profile previously associated with protective immunity against *S. aureus*, their predictive value for field efficacy can only be established through subsequent challenge and field trials.

The adjuvants used here are all based on liposomes supplemented with various immunostimulants. While all four formulations can elicit specific antibodies, two of them have been shown to induce the production of cytokines that are compatible with a cellular immune response that is relevant to *S. aureus* infection: Lip + CpG‐ODN + AG2‐C16 and Lip + Man. The novel lipopeptide exhibited some immunostimulant activity per se, as well as a stabilizing effect on liposome suspension. Additionally, it appears to synergize with CpG‐ODN in inducing IFN‐γ expression. Furthermore, Man supplementation produced a mixed Th1/Th17 response, with the advantage of introducing a single immunostimulant molecule, which may offer a better cost–benefit ratio. The Th17 response plays a crucial role in host defense against extracellular bacterial pathogens by promoting neutrophil recruitment and the stimulation of epithelial antimicrobial defenses at infection sites [39]. This combined response may be advantageous for bacterial clearance through complementary immune mechanisms. Nevertheless, further efficacy studies are needed to determine the most effective adjuvant for the *S. aureus* antigens proposed in this study.

This study has some limitations that should be acknowledged. First, the relatively small number of animals per group (*n* = 5) reflects the exploratory nature of this work, aimed at generating immunogenicity data to guide the selection of promising adjuvant formulations. This sample size may have limited the statistical power to detect subtler differences between formulations, and therefore, the lack of significant differences observed between some formulations should not be interpreted as evidence of true biological equivalence. Second, immune responses were evaluated in healthy, nonchallenged animals, and therefore, the protective capacity of the formulations, as well as their applicability to field conditions, remains to be determined. Future challenge and field trials will be necessary to establish a correlate of protection and confirm clinical efficacy. Third, this study assessed the systemic immune response elicited by subcutaneous immunization in prepartum heifers. However, local antibody responses in milk are directly relevant to *S. aureus* IMIs and should be addressed in future studies involving lactating animals. Despite these limitations, the consistent and significant differences observed between immunized and control groups across multiple immune parameters support the robustness of the immunogenicity findings reported here.

In summary, this study demonstrates that the selected *S. aureus* virulence factors formulated with liposomes combining AG2‐C16, CpG‐ODN, and Man induce a significant humoral response and a Th1‐biased cytokine profile in heifers. These findings provide valuable insights for the development of new vaccine strategies including novel adjuvants in cattle. However, future research is required to optimize these formulations and conduct comprehensive stability testing on those entering translational studies [40].

## Author Contributions

Conceptualization: Cecilia María Camussone and Carolina Melania Isabel Veaute; methodology: Joaquín Cicotello, Guillermo Suarez Archilla, Diego Aliprandi, Agostina Peña, Ivana Gabriela Reidel, María Virginia Zbrun, Marcelo Lisandro Signorini, Sonia Ricotti, and Lourdes Fraile Wirth; formal analysis: Joaquín Cicotello, Camila Miotti, Ana Inés Molineri, Cecilia María Camussone, and Carolina Melania Isabel Veaute; writing–original draft: Joaquín Cicotello, Cecilia María Camussone, and Carolina Melania Isabel Veaute; writing–review and editing: Joaquín Cicotello, Luis Fernando Calvinho, Cecilia María Camussone, and Carolina Melania Isabel Veaute; supervision: Cecilia María Camussone and Carolina Melania Isabel Veaute; project administration: Cecilia María Camussone and Luis Fernando Calvinho; and funding acquisition: Cecilia María Camussone and Luis Fernando Calvinho.

## Funding

This work was supported by the Instituto Nacional de Tecnología Agropecuaria (INTA; 2019‐PD‐E5‐I105‐001; INTA 2023‐PD‐L06‐I116), Agencia Nacional de Promoción Científica y Tecnológica (ANPCyT; PICT‐2017‐2453), Agencia Santafesina de Ciencia Tecnología e Innovación (PEICID‐2021‐147), and Asociación Cooperadora de INTA Rafaela.

## Disclosure

All authors approved the final manuscript, and Carolina Melania Isabel Veaute had full access to the data and assumes full responsibility for its integrity and analysis. The funding sources had no role in study design, data collection, analysis, interpretation, writing, or the decision to submit the paper for publication. Carolina Melania Isabel Veaute affirms that this manuscript is an honest, accurate, and transparent account of the study being reported, that no important aspects of the study have been omitted, and that any discrepancies from the study as planned have been explained.

## Conflicts of Interest

The authors declare no conflicts of interest.

## Supporting Information

Additional supporting information can be found online in the Supporting Information section.

## Supporting information


**Supporting Information** A series of supporting figures is intended to provide additional details on various aspects of this work. Figure S1 shows the vector map of the sequences used to express the antigens. Figure S2 displays an SDS–PAGE and Western blot assay characterizing the obtained recombinant antigens. Figure S3 is a timeline showing the study design. Figure S4 shows the antibody kinetics of each group throughout the study period. Table S1 provides detailed statistical comparisons of antibody levels at each time point studied.

## Data Availability

The datasets generated during and/or analyzed during the current study are available from the corresponding author on reasonable request.
