# Supplementary material for: Novel Liposomal Adjuvants Enhance the Immune Response to Recombinant Staphylococcus aureus Antigens in Dairy Heifers
Source: Vet Med Int. 2026 Sep 23;2026:7845041. doi: 10.1155/vmi/7845041 (PMC13601851; doi:10.1155/vmi/7845041)
Supplement: Supplementary file 1 [file VMI-2026-7845041-s001.zip › Supplementary Figure 2.pdf]

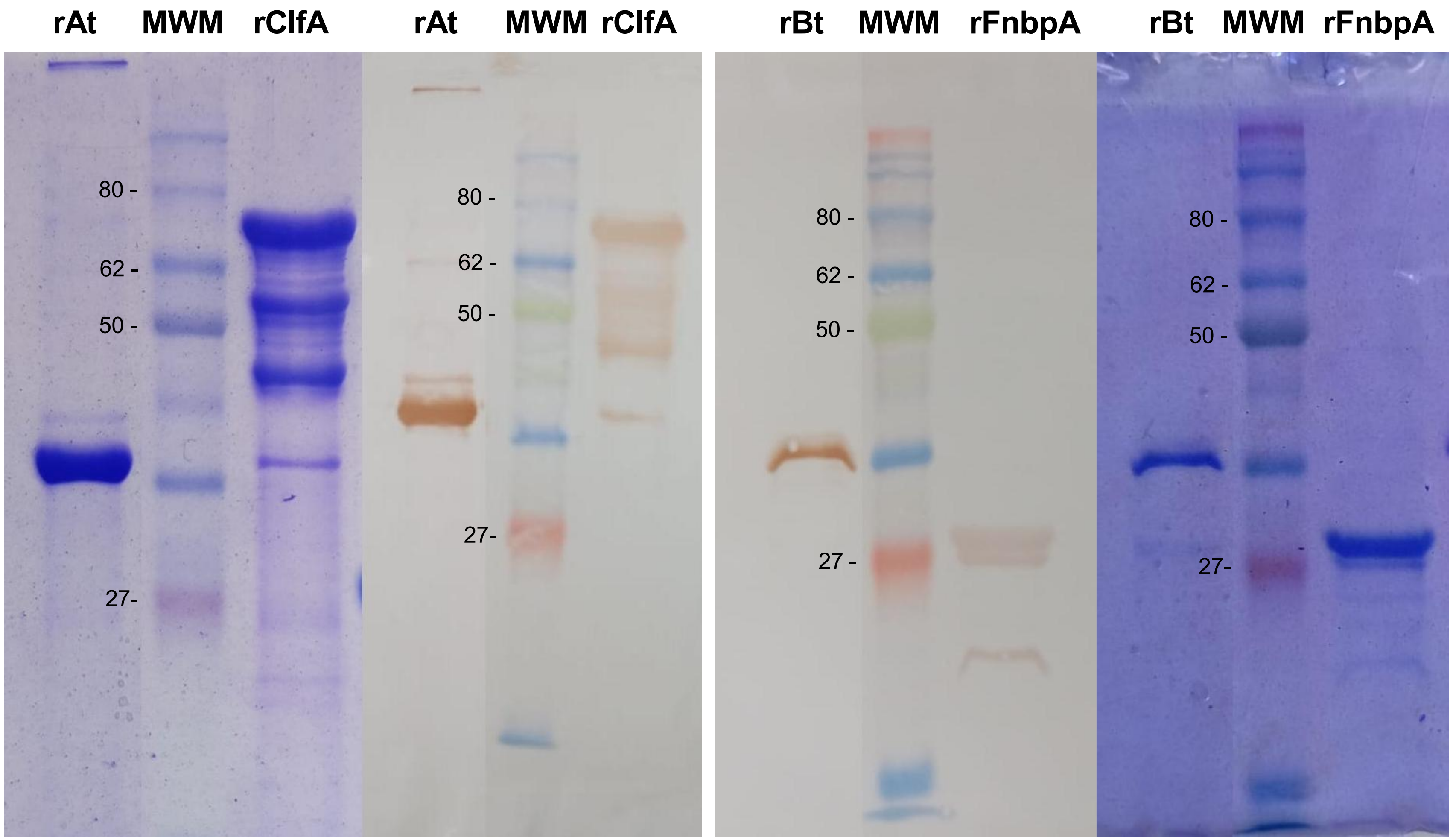

Figure S2A: SDS-PAGE and immunoreactivity of recombinant proteins.

Recombinant proteins were analyzed analyzed by 15% sodium dodecyl sulfate polyacrylamide gel electrophoresis (SDS-PAGE) followed by Coomassie brilliant blue staining. After SDS-PAGE proteins were transferred to nitrocellulose membranes (BioRad Laboratories, CA, USA). Membranes were first incubated with a 5% (w/v) solution of low-fat goat milk powder dissolved in PBS at pH 7.4, and then with heifer sera diluted (1/250) in a 1% (w/v) solution of low-fat goat milk powder dissolved in PBS at pH 7.4. Next, the wells were incubated with anti-bovine IgG conjugated with horseradish peroxidase (Sigma-Aldrich). All incubations were performed for 1 hour at 37 °C. After each step, membranes were washed three times with a 0.05% solution of Tween 20 in PBS. Finally, the reaction was revealed by adding the colorimetric substrate 3,3'-diaminobenzidine tetrahydrochloride (DAB) (Sigma-Aldrich).

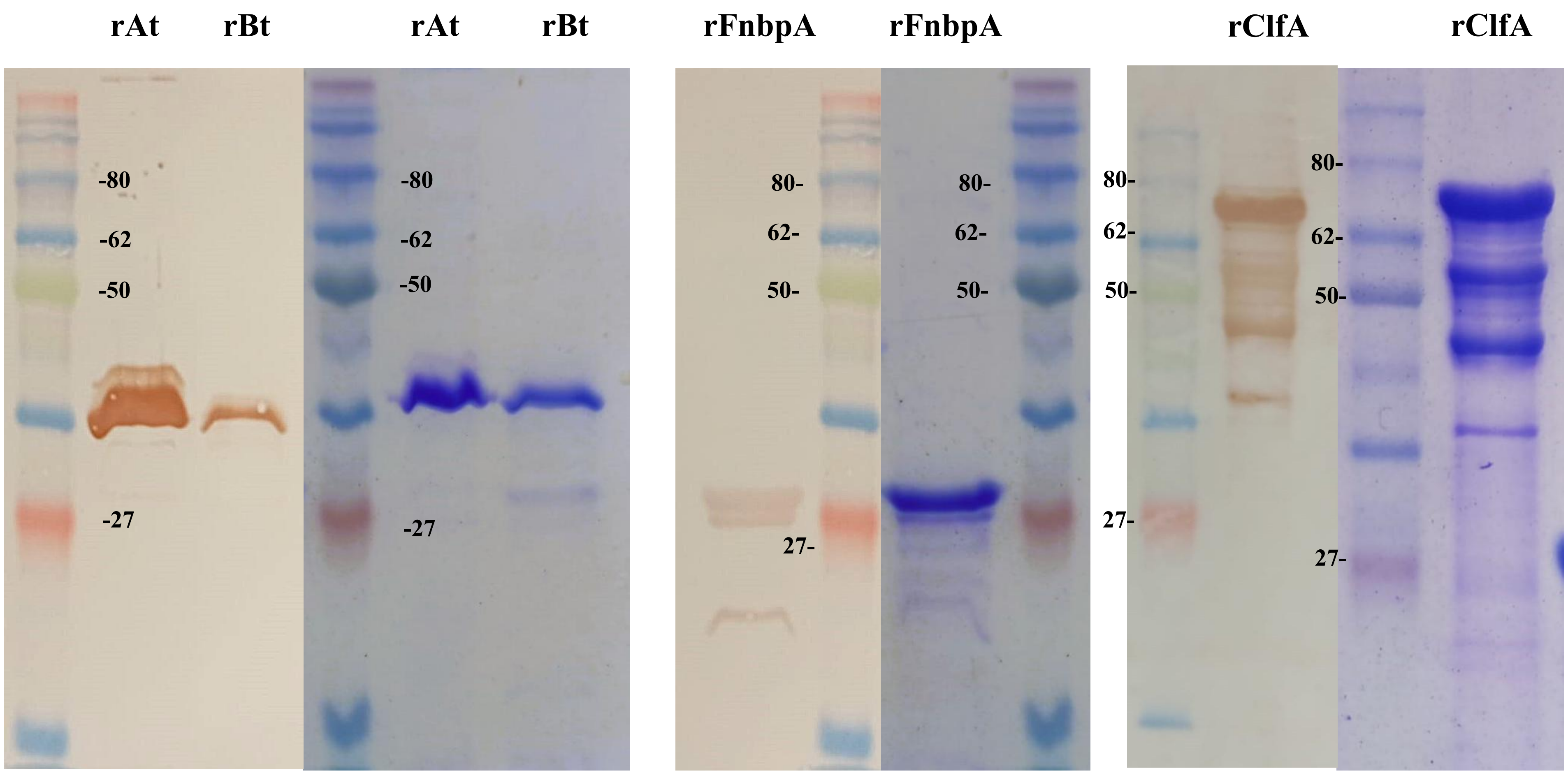

Figure S2-B: SDS-PAGE and identity of recombinant proteins.
Recombinant proteins were analyzed by 15% sodium dodecyl sulfate polyacrylamide gel electrophoresis (SDS-PAGE) followed by Coomassie Brilliant blue staining. After SDS-PAGE proteins were transferred to nitrocellulose membranas (BioRad Laboratories, CA, USA). Membranes were first incubated with a 5% (w/v) solution of low-fat goat milk powder disolved in PBS at pH 7.4, and then with
6x-His Tag Antibody (MA1-21315) in a 1% (w/v) solution of low-fat goat milk poder disolved in PBS at pH 7.4. Next, the wells were incubated with an anti-mouse IgG conjugated with horseradish peroxidase (A4416 , Sigma-Aldrich). All incubations were performed for 1 hour at 37°C. After each step, membranas were washed three times with a 0.05% solution of Tween 20 in PBS. Finally, the reaction was revealed by adding the colorimetric substrate 3,3’-diaminobenzidine tetrahydrochloride (DAB) (Sigma-Aldrich).
