# Supplementary material for: Novel Liposomal Adjuvants Enhance the Immune Response to Recombinant Staphylococcus aureus Antigens in Dairy Heifers
Source: Vet Med Int. 2026 Sep 23;2026:7845041. doi: 10.1155/vmi/7845041 (PMC13601851; doi:10.1155/vmi/7845041)
Supplement: Supplementary file 1 [file VMI-2026-7845041-s001.zip › Supplementary Figure 3.pdf]

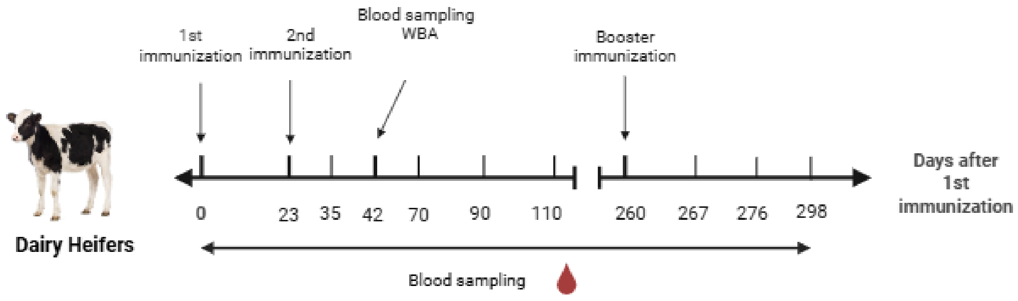

Figure S3. ***Study description***. Heifers were immunized with two doses of immunogen or controls at 0 and 21 days. Eight months after the initiation of the immunization plan, a booster dose was applied. For serologic assays, periodic blood sampling was performed approximately every 15 days from day 0 to day 120, and for 45 days after the booster administration. For Whole Blood Assays, blood sampling was performed 21 days after second dose administration
