# Supplementary material for: Novel Liposomal Adjuvants Enhance the Immune Response to Recombinant Staphylococcus aureus Antigens in Dairy Heifers
Source: Vet Med Int. 2026 Sep 23;2026:7845041. doi: 10.1155/vmi/7845041 (PMC13601851; doi:10.1155/vmi/7845041)
Supplement: Supplementary file 1 [file VMI-2026-7845041-s001.zip › Supplementary Figure 4.pdf]

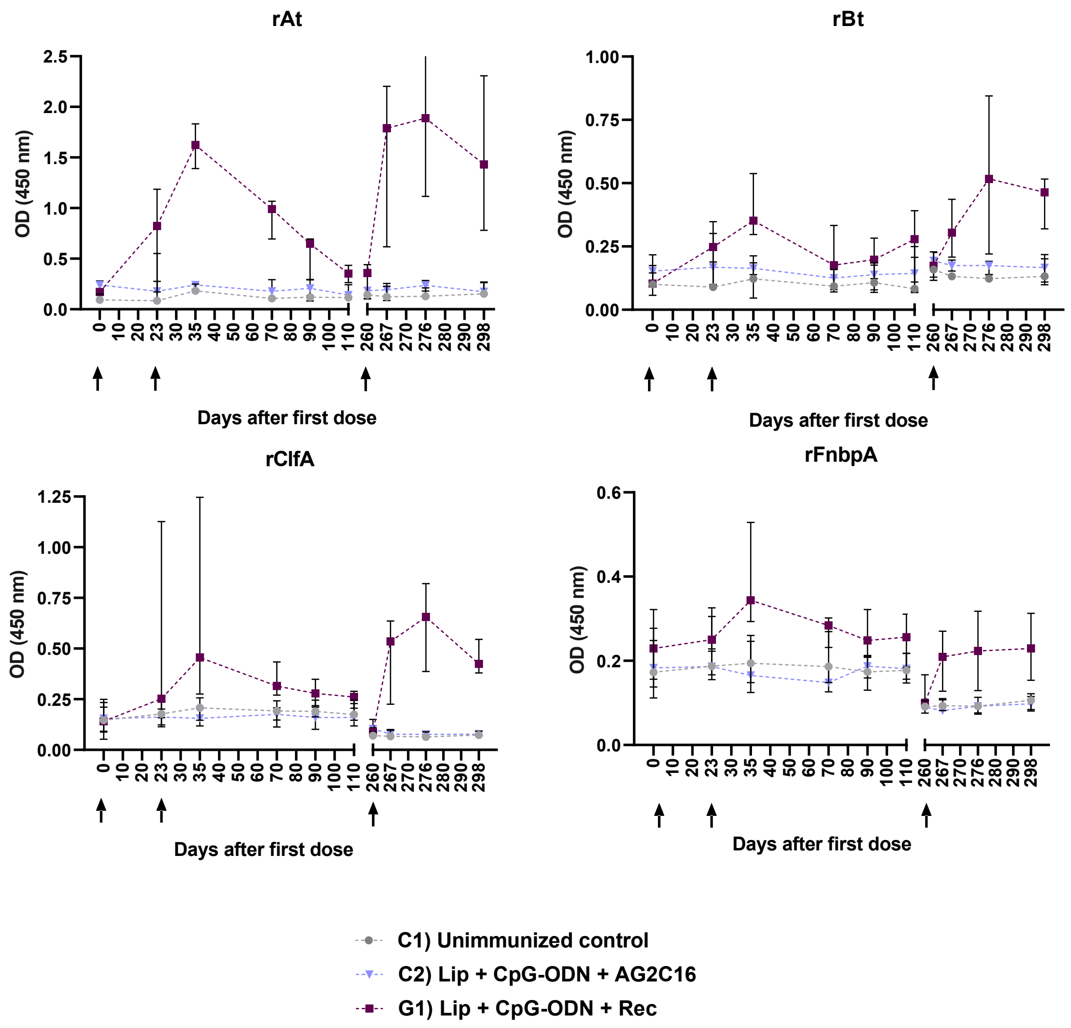

Figure S4a: Specific IgG levels in serum from heifers immunized with Lip+CpG-ODN containing four recombinant antigens (A: anti-rAt, B: anti-rBt, C: anti-rClfA, D: anti-rFnbpA), measured by indirect ELISA. Immunization schedule consisted of two doses and a booster. Sera were diluted 1:1000. Optical density (OD) was measured at 450 nm. Data are presented as median ± interquartile range (IQR) for each group. Arrows indicate the days of vaccination

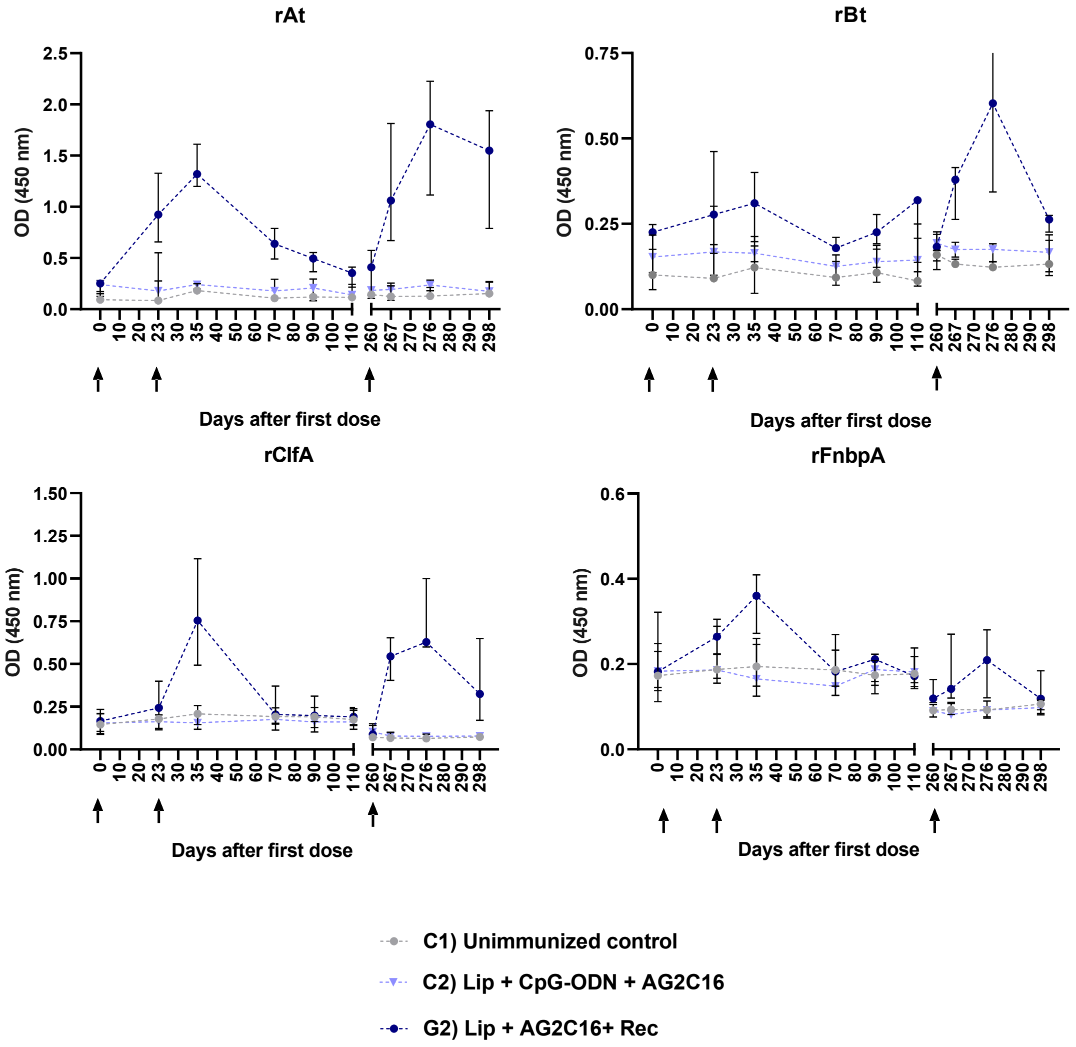

Figure S4b: Specific IgG levels in serum from heifers immunized with Lip+AG2C16 containing four recombinant antigens (A: anti-rAt, B: anti-rBt, C: anti-rClfA, D: anti-rFnbpA), measured by indirect ELISA. Immunization schedule consisted of two doses and a booster. Sera were diluted 1:1000. Optical density (OD) was measured at 450 nm. Data are presented as median ± interquartile range (IQR) for each group. Arrows indicate the days of vaccination

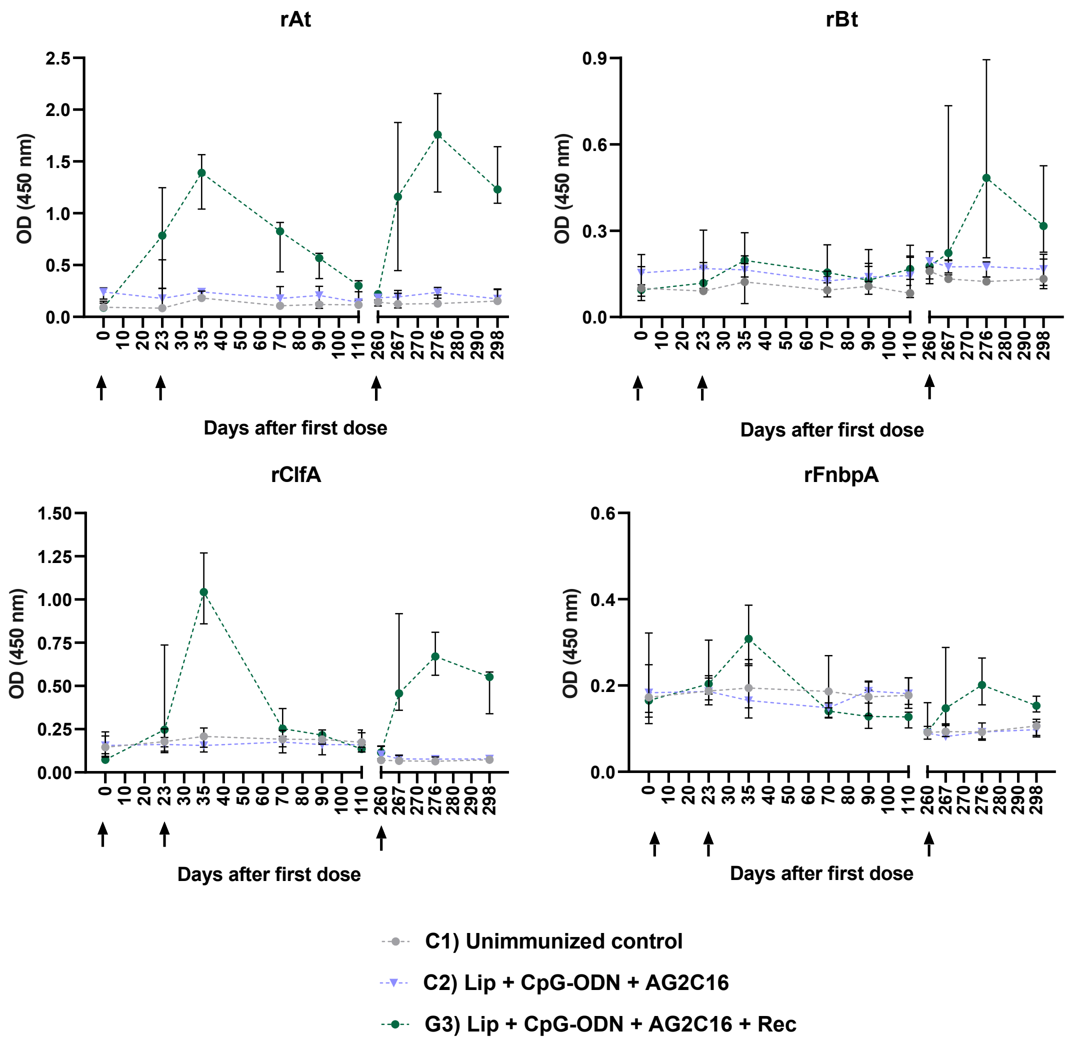

Figure S4c: Specific IgG levels in serum from heifers immunized with Lip+CpG-ODN+AG2C16 containing four recombinant antigens (A: anti-rAt, B: anti-rBt, C: anti-rClfA, D: anti-rFnbpA), measured by indirect ELISA. Immunization schedule consisted of two doses and a booster. Sera were diluted 1:1000. Optical density (OD) was measured at 450 nm. Data are presented as median ± interquartile range (IQR) for each group. Arrows indicate the days of vaccination

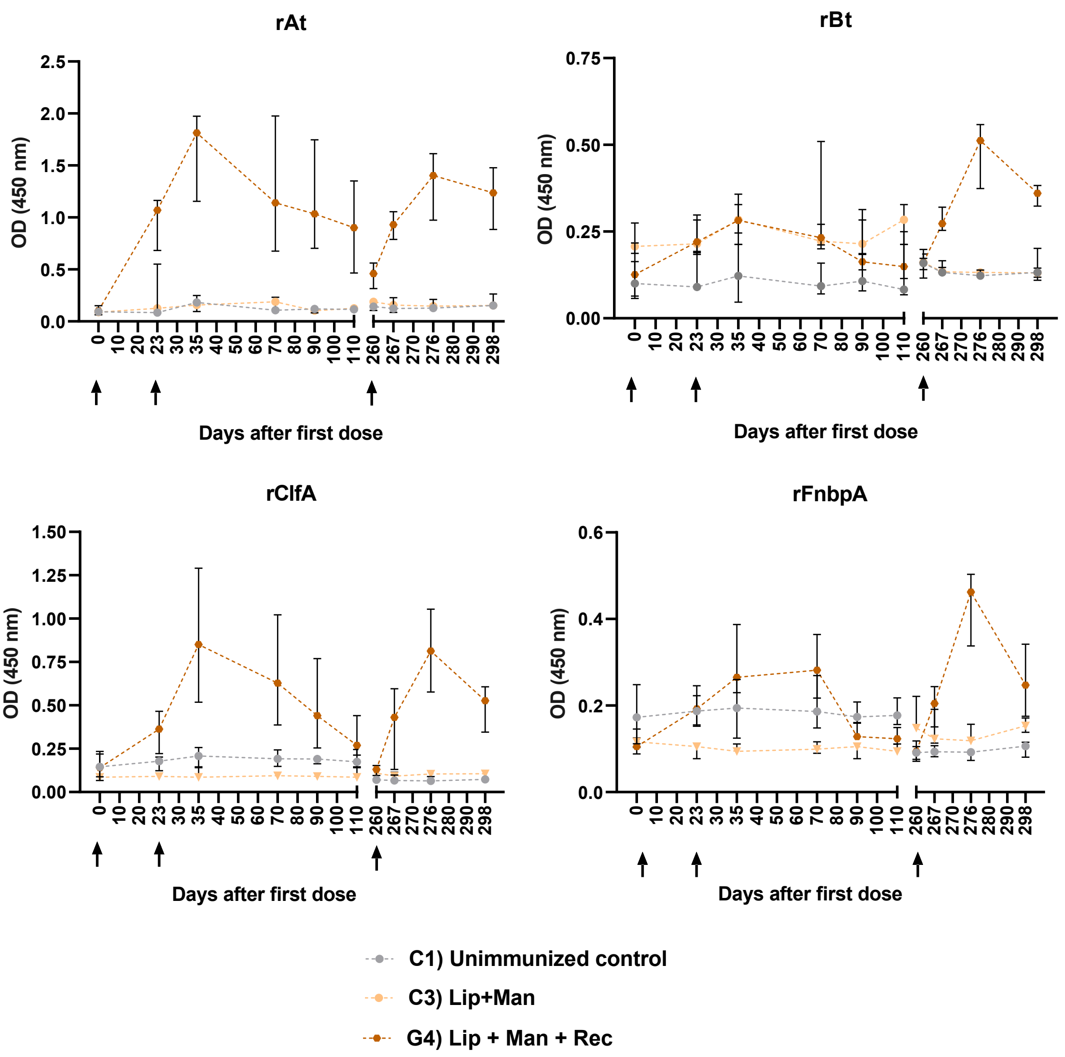

Figure S4d: Specific IgG levels in serum from heifers immunized with Lip+Man containing four recombinant antigens (A: anti-rAt, B: anti-rBt, C: anti-rClfA, D: anti-rFnbpA), measured by indirect ELISA. Immunization schedule consisted of two doses and a booster. Sera were diluted 1:1000. Optical density (OD) was measured at 450 nm. Data are presented as median ± interquartile range (IQR) for each group. Arrows indicate the days of vaccination
