# Supplementary material for: Novel Liposomal Adjuvants Enhance the Immune Response to Recombinant Staphylococcus aureus Antigens in Dairy Heifers
Source: Vet Med Int. 2026 Sep 23;2026:7845041. doi: 10.1155/vmi/7845041 (PMC13601851; doi:10.1155/vmi/7845041)
Supplement: Supplementary file 1 [file VMI-2026-7845041-s001.zip › TablaS1.docx]

| Table S1A | G1: Lip+CpG-ODN+Rec | | | | G2: Lip+AG2C16+Rec | | | | G3: Lip+ CpG-ODN+AG2C16+Rec | | | | G4: Lip + Man+Rec | | | |
| --- | --- | --- | --- | --- | --- | --- | --- | --- | --- | --- | --- | --- | --- | --- | --- | --- |
| Days | **rAT** | **rBT** | **rClfA** | **rFnbpA** | **rAT** | **rBT** | **rClfA** | **rFnbpA** | **rAT** | **rBT** | **rClfA** | **rFnbpA** | **rAT** | **rBT** | **rClfA** | **rFnbpA** |
| 0 | 0.158^ns^ | 0.115^ns^ | 0.149^ns^ | 0.219^ns^ | 0.209^ns^ | 0.187^ns^ | 0.158^ns^ | 0.186^ns^ | 0.100^ns^ | 0.092^ns^ | 0.084^ns^ | 0.151^ns^ | 0.100^ns^ | 0.116^ns^ | 0.142^ns^ | 0.113^ns^ |
| 23 | 0.707^ns^ | 0.226^ns^ | 0.564^ns^ | 0.271^*a^ | 0.978^ns*b^ | 0.305^ns^ | 0.297^ns^ | 0.257^*a^ | 0.765^ns^ | 0.131^ns^ | 0.403^ns^ | 0.199^ns^ | 0,952^ns**c^ | 0.349^ns^ | 0.347^ns**c^ | 0.195^ns**c^ |
| 35 | 1.614^**ab^ | 0.404^**ab^ | 0.699^*ab^ | 0.397^*ab^ | 1.388^*ab^ | 0.301^ns*b^ | 0.794^*ab^ | 0.344^*ab^ | 1.321^*ab^ | 0.231^ns^ | 1,060^**ab^ | 0.316^ns^ | 1,614^**ac^ | 0.297^ns^ | 0.893^*a**c^ | 0.299^ns**c^ |
| 70 | 0.902^**ab^ | 0.202^ns^ | 0.344^ns**b^ | 0.270^ns*b^ | 0.639^ns^ | 0.182^ns^ | 0.250^ns^ | 0.180^ns^ | 0.703^ns*b^ | 0.178^ns^ | 0.294^ns*b^ | 0.141^ns^ | 1.289^**ac^ | 0.334^**a^ | 0.688^**ac^ | 0.288^ns**c^ |
| 90 | 0.349^ns*b^ | 0.180^ns^ | 0.282^ns^ | 0.263^ns^ | 0.321^ns*b^ | 0.200^ns^ | 0.215^ns^ | 0.191^ns^ | 0.264^ns*b^ | 0.168^ns^ | 0.200^ns^ | 0.141^ns^ | 0,906^**ac^ | 0.213^ns^ | 0.497^*ac^ | 0.139^ns^ |
| 110 | 0.353^*a^ | 0.241^ns^ | 0.250^ns^ | 0.249^ns^ | 0.354^ns^ | 0.249^ns^ | 0.189^ns^ | 0.186^ns^ | 0.348^ns^ | 0.170^ns^ | 0.141^ns^ | 0.121^ns^ | 1.035^**ac^ | 0.160^ns^ | 0.315^ns*c^ | 0.128^ns^ |

| Table S1B | G1: Lip+ CpG-ODN +Rec | | | | G2: Lip+AG2C16+Rec | | | | G3: Lip+ CpG-ODN+AG2C16+Rec | | | | G4: Lip+Man+Rec | | | |
| --- | --- | --- | --- | --- | --- | --- | --- | --- | --- | --- | --- | --- | --- | --- | --- | --- |
| Days | **rAT** | **rBT** | **rClfA** | **rFnbpA** | **rAT** | **rBT** | **rClfA** | **rFnbpA** | **rAT** | **rBT** | **rClfA** | **rFnbpA** | **rAT** | **rBT** | **rClfA** | **rFnbpA** |
| 260 | 0.289^ns^ | 0.177^ns^ | 0.092^ns^ | 0.124^ns^ | 0.366^ns^ | 0.180^ns^ | 0.105^ns^ | 0.131^ns^ | 0.198^ns^ | 0.161^ns^ | 0.116^ns^ | 0.117^ns^ | 0.443^*a^ | 0.173^ns^ | 0.125^ns^ | 0.094^ns^ |
| 267 | 1.486^**a*b^ | 0.318^ns^ | 0.451^ns*b^ | 0.201^*ab^ | 1.205^*ab^ | 0.346^*a^ | 0.531^*ab^ | 0.184^ns^ | 1.162^*ab^ | 0.417^*ab^ | 0.602^*ab^ | 0.189^ns^ | 0.924^*a**c^ | 0.284^ns**c^ | 0.376^ns^ | 0.198^*ac^ |
| 276 | 1.824^*ab^ | 0.529^*ab^ | 0.613^*ab^ | 0.223^ns^ | 1.698^*ab^ | 0.610^**a*b^ | 0.765^*ab^ | 0.202^ns^ | 1.696^*ab^ | 0.536^ns*b^ | 0.683^*ab^ | 0.207^ns^ | 1.315^*ac^ | 0.476^ns**c^ | 0.813^**ac^ | 0.428^**ac^ |
| 298 | 1.522^**a*b^ | 0.472^**ab^ | 0.454^*ab^ | 0.232^*ab^ | 1.401^*ab^ | 0.252^ns^ | 0.393^ns^ | 0.134^ns^ | 1,342^*ab^ | 0.363^ns*b^ | 0.478^*a*b^ | 0.156^ns^ | 1.193^*a^ | 0.354^*a**c^ | 0.486^**ac^ | 0.256^**a*c^ |

**Table S1**. Specific IgG levels in serum from heifers immunized with different formulations containing four recombinant antigens (rAt, rBt, rClfA, rFnbpA), measured by indirect ELISA and expressed as OD values. Immunization schedule consisted of two doses and a booster. Sera were diluted 1:1000. References: Superscript letters indicate significant differences: a: compared with the Unimmunized control, b: compared with Lip+CpG-ODN+AG2-C16, and c: compared with the Lip+Man control. A Kruskal-Wallis statistical analysis was performed to compare differences between groups, followed by Dunn's correction for multiple comparisons. The median of animal sera from each group is shown. ns: not significant; *p < 0.05; **p < 0.01; ***p < 0.001; ****p < 0.0001.
