# Supplementary material for: Novel Liposomal Adjuvants Enhance the Immune Response to Recombinant Staphylococcus aureus Antigens in Dairy Heifers
Source: Vet Med Int. 2026 Sep 23;2026:7845041. doi: 10.1155/vmi/7845041 (PMC13601851; doi:10.1155/vmi/7845041)
Supplement: Supplementary file 1 [file VMI-2026-7845041-s001.zip › Supplementary Figure 1.pdf]

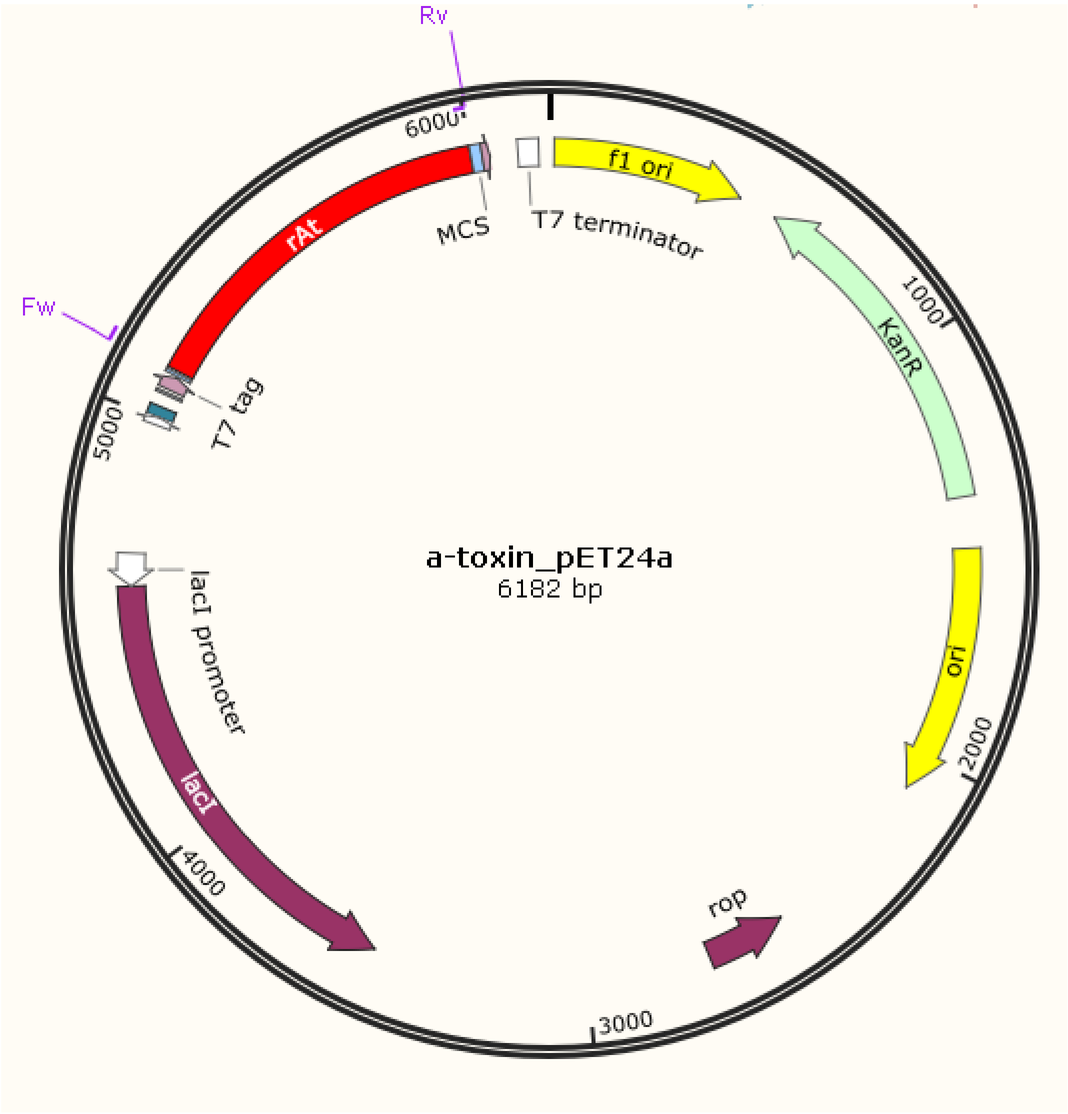

Figure S1a: Expression vector map for rA-toxin

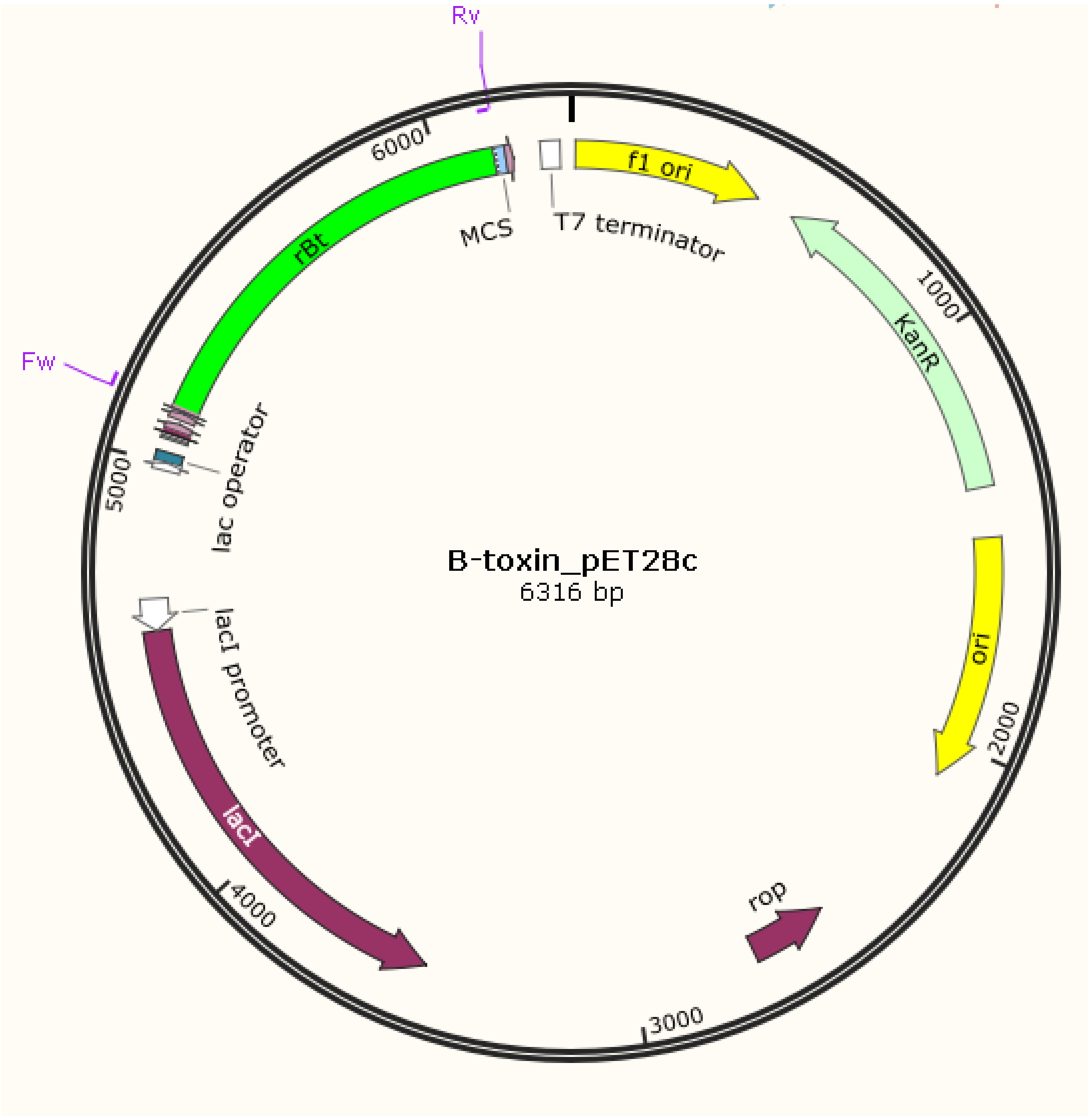

Figure S1b: Expression vector map for rB-toxin

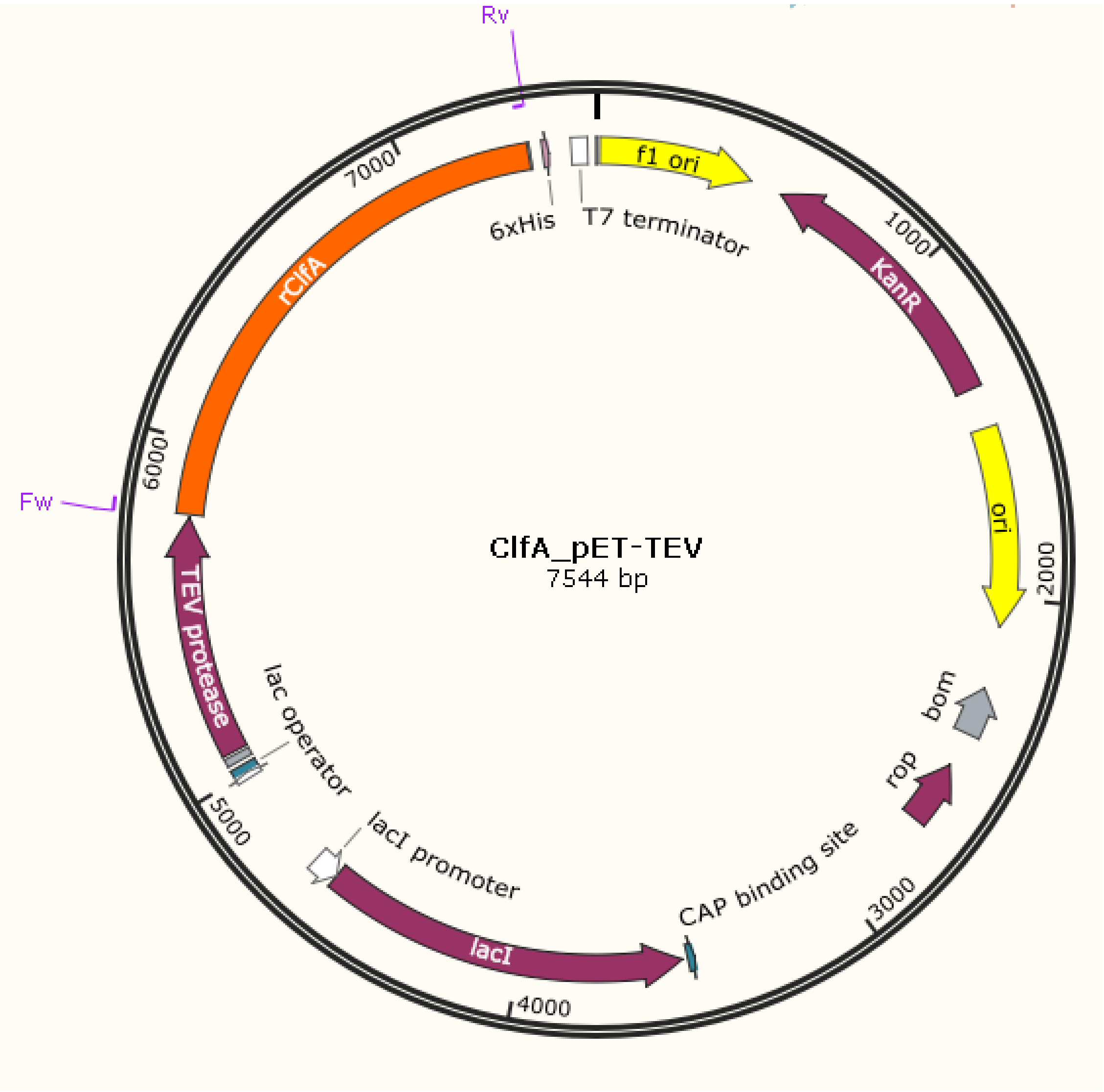

Figure S1c: Expression vector map for rClumping Factor A

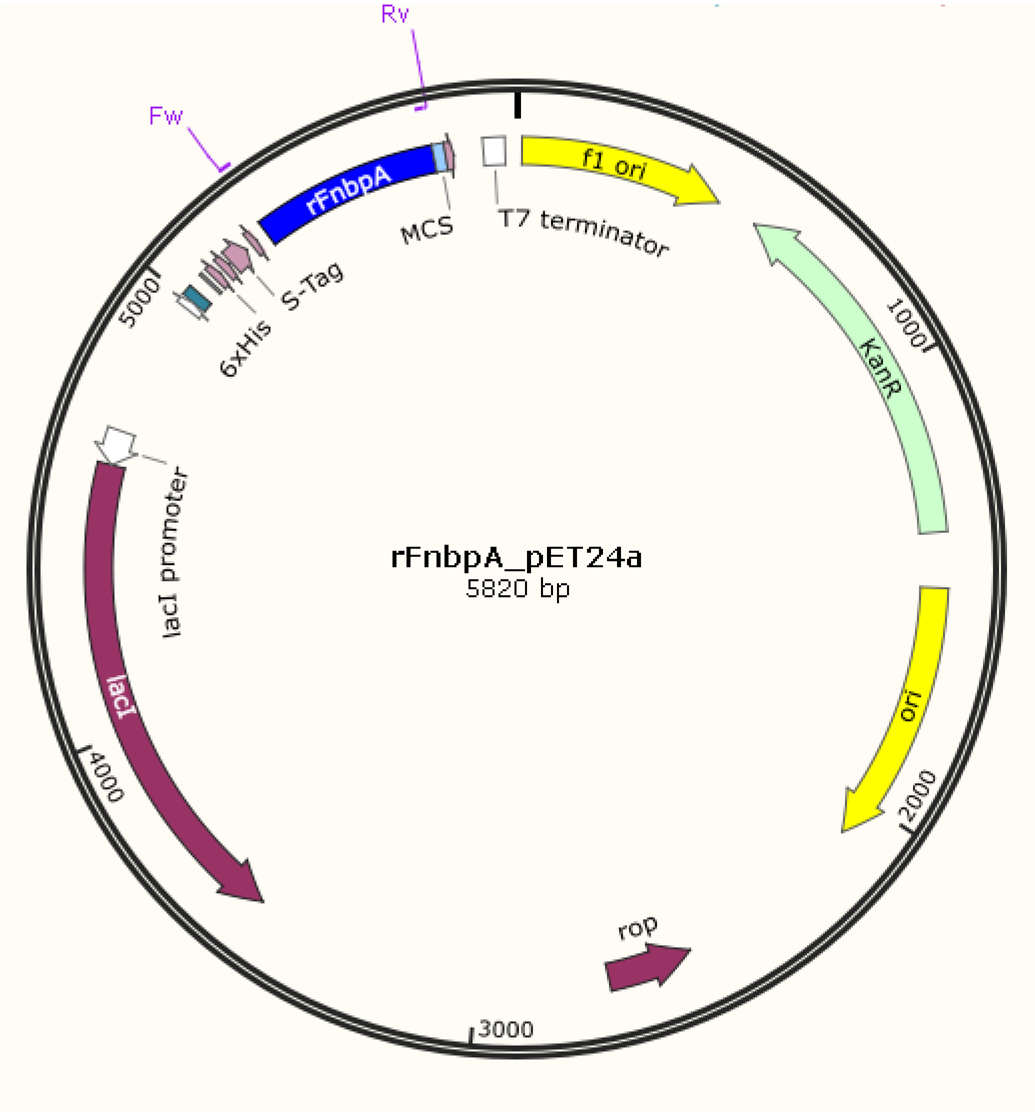

Figure S1d: Expression vector map for rFibronectin Binding Protein A
